# Development and validation of an AI-enabled oral score using large-scale dental data

**DOI:** 10.1038/s41598-025-07484-7

**Published:** 2025-07-01

**Authors:** Sri Kalyan Yarlagadda, Navid Samavati, Mina Ghorbanifarajzadeh, Vlada Levinta, Alireza Sojoudi, Wardah Inam, Teresa A. Dolan

**Affiliations:** Overjet, Inc., 2093 Philadelphia Pike #9194, Claymont, DE 19703 USA

**Keywords:** Dental conditions, Dental public health

## Abstract

This research introduces Oral Score Basic (OS-B), a novel Artificial Intelligence (AI) derived methodology designed to provide a comprehensive, objective assessment of individual teeth and overall oral health, initially focused on dental conditions. Leveraging data from more than 340,000 patients across 2,558 U.S. dental practices, OS-B combines radiographic findings and periodontal probing depths with a treatment probability-weighted cost function to quantify the severity of dental conditions. The OS-B score aims to address limitations in prior oral health scoring systems by incorporating nuanced clinical data accounting for disease severity, and providing a scalable, data-driven approach to measuring oral health. This score was developed using Overjet’s FDA-cleared AI platform, which detects dental conditions using bitewing and periapical radiographs, providing a detailed analysis of each tooth. OS-B’s effectiveness was validated by demonstrating a strong correlation between tooth scores and treatment costs, surpassing the predictive power of previous scoring systems. This research presents a foundational framework for AI-enabled oral health scoring, with potential applications in value-based care, population risk analysis, and consumer health management. Future iterations may expand to include additional dimensions of oral health beyond clinical conditions such as risk factors and measures of oral function and esthetics, further enhancing the score’s public health and clinical utility and patient engagement.

## Introduction

Oral health is a critical component of overall health and well-being; yet quantifying it comprehensively has remained a challenge. Over the past five decades, numerous oral health scores have been developed to summarize oral health status and to measure the impact of healthcare interventions. Notable examples include the work of Nikkias et al.^1,2^, the Index of Oral Health Status by Marcus et al.^3^, the Oral Health Index published by Burke and Wilson^4^ that was later modified and developed by Denplan (Winchester, UK) and renamed the Oral Health Score^5^. Self-reported measures of oral health and oral health related quality of life such as the OHIP-5^6^, and the GOHAI^7^ have been developed and extensively validated. However, these measures are based on patient reports and have several important limitations including recall bias, lack of clinical specificity, and limited sensitivity to change following treatment interventions. More recently, commercial products such as Previser have emerged as an evidence-based risk score for oral diseases^8^. While these previous efforts have been valuable, they are constrained by limited sample sizes and often rely on binary disease classifications, failing to capture the nuanced complexity of oral health conditions. The advent of artificial intelligence (AI) and advanced computer vision techniques powered by deep learning now presents an unprecedented opportunity to revolutionize oral health assessment.

Table 1 provides a comparative overview of prior methodologies to create an oral health outcome measure, highlighting both their strengths and limitations in relation to a new proposed methodology that utilizes AI derived clinical findings and cost-weighting in a large U.S. national data set (OS-B). While existing tools like the Oral Health Status Index (OHSI), the 5-item Oral Health Impact Profile (OHIP-5), and traditional epidemiological measures—such as the Decayed, Missing, and Filled Teeth (DMFT) index and the Community Periodontal Index (CPI)—serve specific purposes, they do not offer a comprehensive measure of oral health status or effectively predict treatment needs and related costs. These indices typically rely on subjective evaluations, whereas the proposed OS-B leverages AI-driven detection and a treatment probability-weighted cost function, resulting in a more precise and clinically relevant evaluation.

**Table 1:** Comparative overview of oral health indices: strengths, limitations, and performance relative to the proposed ai enabled composite oral score.

Criteria	Marcus index (OHSI)	The 5-item oral health impact profile (OHIP-5)	Traditional epidemiological measures (DMFT, CPI, etc.)	OS-B (AI-based oral health assessment with cost-weighting)
Primary assessment method	Composite scoring of oral health indicators	Patient-reported questionnaire on oral health-related quality of life (OHRQoL)	Clinical examination of dental and periodontal status	AI-driven analysis of radiographic and clinical data
Objectivity & consistency	Objective, but method-dependent	Subjective; relies on patient responses	Objective, but examiner-dependent	Highly objective—AI ensures standardized, automated evaluations across large datasets
Disease severity categorization	Provides a single index score	Limited; ordinal scale from 0–4	Basic severity grading (e.g., DMFT counts decayed teeth)	Advanced, using AI to quantify severity trends
Predictive power for treatment needs	Low—focuses on oral function rather than clinical disease progression	Low; does not predict treatment needs	Low; primarily records current status	Strong—correlation coefficient (-0.441), representing a 200% improvement over OHSI
Coverage of oral health factors	Assesses functional limitations (e.g., chewing ability) rather than clinical conditions	Focuses on quality of life impacts	Focuses on caries, missing teeth, and periodontal disease	More comprehensive—assesses teeth, bone levels, treatment history, and cost implications
Periodontal assessment	None—does not directly assess periodontal health	None	Assesses periodontal pockets (CPI)	Limited—assesses interproximal bone levels but excludes soft tissue and functional measures
Cost consideration	None	None	None	Uses CDT-coded treatment costs to weight disease severity
Real-time decision support	None	None	None	Provides real-time, AI-assisted insights
Personalization for patients	Population-based analysis	Generalized OHRQoL assessment	Population-based analysis	Patient-level scoring with potential for individualized monitoring and intervention planning
Limitations	Limited sensitivity to disease severity	Subjective, lacks disease-specific data	Lacks predictive insights, does not capture patient experience	Relies on radiographic data from patients with dental visits; does not account for uncompleted treatments, soft tissue conditions, or patient-reported outcomes

The development of a more sophisticated oral health score is imperative, driven by several significant healthcare trends. The ongoing transformation from fee-for-service to value-based care models necessitates robust outcome measures. An AI-derived oral health score could precisely quantify changes in oral health related to clinical interventions, enabling more accurate assessment of care effectiveness. Concurrently, the shift in dental practice modality, with an increasing rate of dentists affiliating with dental support organizations and practicing in groups^9^, provides an opportunity to measure and monitor services provided and their impact on health status. Moreover, the growing consumer interest in health monitoring and management calls for accessible tools that empower individuals. A consumer-friendly oral health score could play a crucial role in early detection and prevention, potentially reducing the need for invasive and costly treatments. Gamification of such a score could further engage and motivate consumers to better manage their oral health. Finally, private or public payers of care would benefit from an objective clinical outcome measure that could be utilized in population risk analysis, plan design, and provider network assessment. These factors collectively underscore the need for a comprehensive, AI-driven oral health score that can serve multiple stakeholders in the healthcare ecosystem.

This research aims to address these needs by developing and validating a novel, AI-enabled composite oral score—Oral Score Basic (OS-B)—that overcomes the limitations of prior oral health scoring systems. Our methodology leverages large-scale clinical data from 2558 dental practices and more than 343,000 patients across all 50 U.S. states, representing one of the most comprehensive and geographically diverse datasets ever used in dental research. OS-B uses objective, quantifiable measures to assess individual tooth health, combining radiographic findings and periodontal measurements with a treatment probability-weighted cost function. This approach moves beyond binary disease classification by capturing disease severity—for example, quantifying caries using the relationship between DMFP and treatment cost. Tooth-level scores are generated using FDA-cleared deep learning models, which provide consistent and scalable assessments far more reliable than self-reported or examiner-dependent evaluations. The individual tooth scores are then averaged to compute a patient-level oral score. Because this initial version focuses specifically on dental health indicators, we refer to it as Oral Score – Basic (OS-B). This foundational research establishes a framework that can be expanded to include additional oral health dimensions such as function, esthetics, and patient-reported outcomes, aligning with the World Health Organization’s holistic definition of oral health^10^.We hypothesize that AI-driven analysis can yield a more consistent, objective, and scalable approach to oral scoring based on the most comprehensive and accurate data available and tested this hypothesis by comparing the OS-B scores to the Marcus et. Al OHSI^3^. Our study utilizes AI analysis of dental radiographs from an unprecedented sample of 343,297 patients across 2,558 dental practices in the United States. This extensive dataset allows for a more nuanced and comprehensive assessment of dental conditions than ever before possible.

By developing this innovative scoring system, we aim to provide a valuable tool for clinicians, researchers, and policymakers to better understand, monitor, and improve oral health outcomes. Additionally, with further development and validation, the OS-B has the potential to empower consumers in managing their oral health, ultimately contributing to improved overall health and reduced healthcare costs.

## Methods

**Fig. 1:**
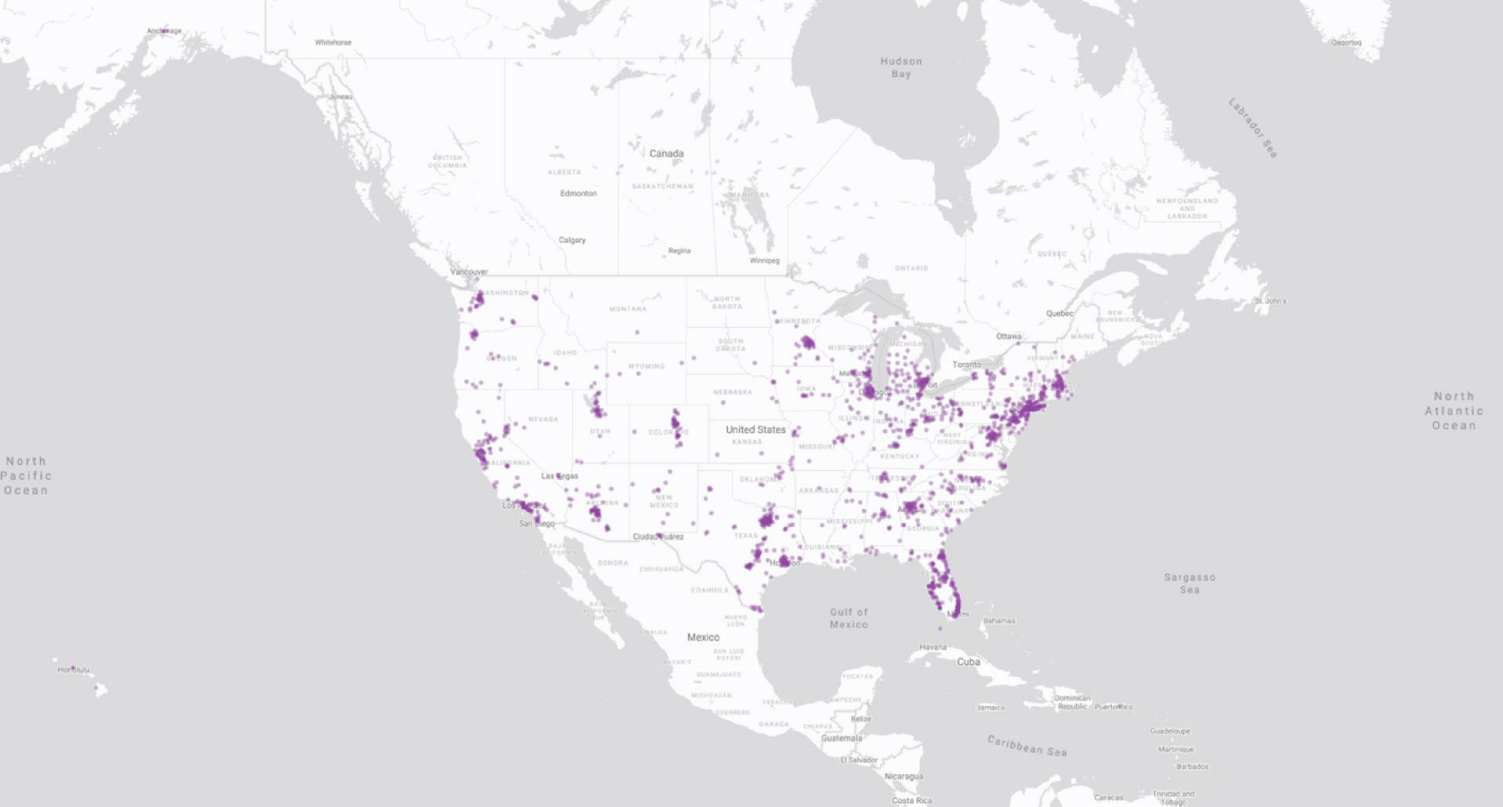
Geographic distribution of the 2558 dental practices whose data were used to develop the OS-B.

The OS-B is built using data from 2,558 dental practices across the United States who used the Overjet, Inc. Practice Application^11^ and includes data from 321,530 adult patients who were 21 years of age or older (Figure 1). All patient data were deidentified in accordance with HIPAA guidelines to ensure confidentiality. These practices are located in every U.S. state as well as Puerto Rico.

The development of the OS-B included defining the clinical components of the score, developing a test dataset and subsets, and developing a novel treatment probability weighted cost-function to calculate a weighted individual tooth score from each of the patient’s 28 permanent teeth, excluding third molars. The adult human dentition typically includes up to 32 teeth, including four third molars (wisdom teeth). Contemporary dental public health research increasingly adopts 28-tooth frameworks for population-level studies. This methodology uses the 28 tooth framework in order to determine a more consistent and comparable metric across diverse demographic groups, minimizing confounding variables associated with third molar variability. We acknowledge that third-molars can impact overall health, particularly in the context of periodontal disease, and future research should further explore the use of the 32- vs. the 28-tooth framework in the score calculation. For the purposes of this study, individual tooth scores for the patient’s 28 permanent teeth were then averaged into a mouth-level summary score called OS-B. Once constructed, we conducted a preliminary validation of OS-B on the test dataset and compared the OS-B to the Marcus et. al OHSI^3^.

The clinical condition of the 28 permanent teeth was assessed using findings from the Overjet AI platform and its proprietary, FDA-cleared Machine Learning Algorithms (MLA) along with periodontal probing depth data from patient electronic records. Overjet’s AI models for detecting and segmenting caries, calculus, periapical radiolucencies (PARL), margin discrepancies, and existing restorations—including fillings, crowns, root canal-treated (RCT) teeth, and implants—are all based on a proprietary Convolutional Neural Network architecture designed to perform object detection and segmentation on dental radiographs. The architecture includes a CNN backbone based on ResNet with Feature Pyramid Network (FPN) for feature extraction, a Region Proposal Network (RPN) to generate candidate object regions, and a final stage that predicts bounding box locations, instance masks, and keypoints as needed. Table 2 presents the standalone sensitivity and specificity of each model used in this research.

**Table 2:** Standalone sensitivity and specificity of Overjet’s AI models for dental condition detection and segmentation.

FDA clearance number	Model name	Reported stand alone sensitivity and specificity
K210187^12^	Bone level	Precision greater than 0.5 mm Sensitivity > 88%, Specificity > 95%
K233590^13^	Restorations	Sensitivity > 80%, Specificity > 98%
K231678^14^	PARL	Sensitivity > 88%, Specificity > 84%
K233738^15^	Caries	Sensitivity > 83%, Specificity > 97%
K220928^16^	Calculus	Sensitivity > 73%, Specificity > 99%

**Fig. 2:**
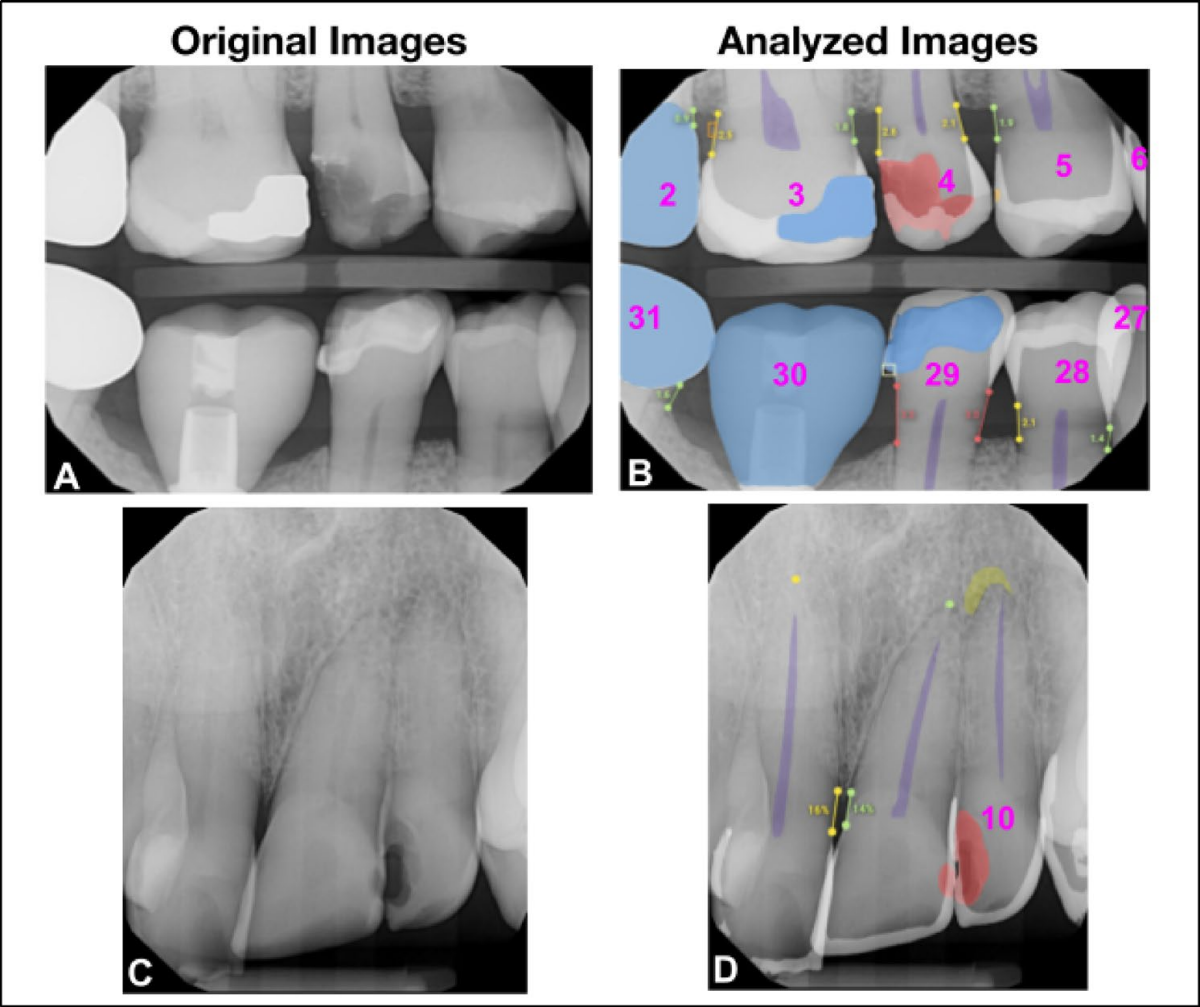
This figure illustrates the clinical findings on dental radiographs as they appear in their original state and as analyzed by the Overjet AI platform. Images (**A**) and (**C**) are the original radiographs; images (**B**) and (**D**) are analyzed radiographs by the Overjet AI platform. Image B has segmentations in white to represent enamel, purple for pulp, blue for restorations including implant restorations, red for caries, marginal discrepancies in yellow box, calculus in an orange box, and millimeter bone level measurements in green, yellow and red corresponding to the value measured. Tooth numbers are presented in pink. Image (**D**). In addition to identifying caries and measuring bone levels, this radiograph includes a PARL, indicated as a yellow-green crescent shape at the apex area of tooth number 10.

**Fig. 3:**
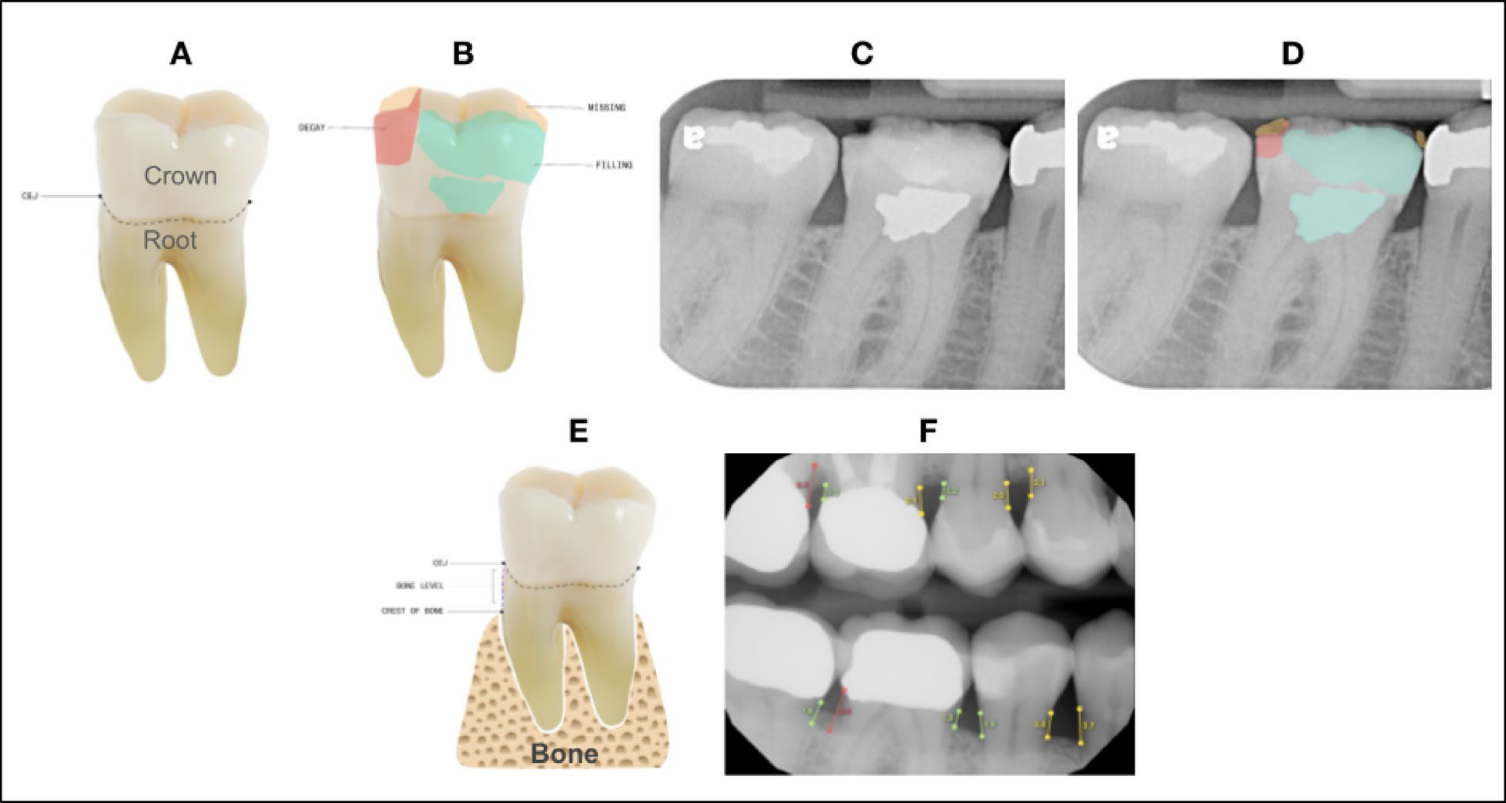
Top ROW*:* The teeth images (**A**) and (**B**) are used to illustrate 2-dimensional segmentations similar to the radiographs (**C**) and (**D**). From the top left, in image (**A**) illustrates the location of the CEJ on the tooth. The portion of the tooth identified as above or coronal to the dotted line is defined as the coronal portion of the tooth, and the area of the tooth below or apical to the CEJ is considered the root portion of the tooth. Image (**B**) depicts in 2 dimensions how the tooth is segmented to calculate DMFP by identifying the coronal portion of the tooth that is decayed (red), missing (orange), and filled (green). Image (**C**) shows a standard periapical radiograph without AI generated predictions. Image (**D**) shows the AI-analyzed image illustrating the decayed, missing and filled segmentations on this radiograph. The DMFP calculation for tooth number 30 is 0.71. *Bottom Row:* Image (**E**) illustrates the anatomical landmarks that are used to measure the interproximal alveolar bone level: CEJ and crest of bone. The distance between these two points is the reported bone level (BL). This measurement is analyzed on the mesial and distal of each tooth on the radiograph and can be seen on the bone level image (**F**).

Overjet’s algorithms detect and segment clinical conditions on bitewing and periapical radiographs, and Figs. 2 and 3 provide examples of how these clinical findings are noted on dental radiographs.

The dental conditions analyzed by the Overjet AI platform include:

1. Tooth status as either present, missing, or a root tip, which is defined as a tooth with more than 95 percent of the anatomical crown either missing or decayed.
2. Radiolucencies on the tooth structure indicative of demineralization and/or dental caries.
3. The type and extent of dental restorations on an individual tooth including radiographic evidence of full and partial coverage crowns, fillings, root canals, and/or the presence of a dental implant in place of the tooth.
4. The percentage of the tooth’s coronal tooth structure that is decayed, missing and/or filled, calculated by the Overjet platform as the Decayed, Missing, and/or Filled Proportion (DMFP).
5. Interproximal alveolar bone levels (ABL) measured in millimeters from the cemento-enamel junction (CEJ) to the most apical crest of the interproximal alveolar bone, as an indicator of the tooth’s periodontal status.
6. Interproximal calculus on cementum for each tooth on both bitewing and periapical radiographs, scored as either absent or present.
7. Periapical Radiolucencies (PARL) on periapical radiographs that may or may not be associated with an endodontic root filling. PARL is scored as either absent or present.
8. Margin discrepancy (MD) where a full or partial coverage crown or filling has a defective margin, an over contoured or under contoured restoration, or an overhang where a restorative material extends beyond or over the margin apically. MD is scored as either absent or present. Note that this feature of the Overjet AI platform is not currently FDA cleared but was included in the analysis because it adds information about the quality of existing restorations.

### AI model performance and training process

As part of the data cleaning and preprocessing pipeline, all patient and clinic data extracted from practice management systems (PMS) were assigned unique identifiers to prevent cross-association of information. Radiographs were matched to appointment dates, and any radiographs lacking an associated appointment date, as well as patients without documented age, were excluded from the dataset. Additionally, data related to proposed dental treatment plans or delivered treatments were linked to the corresponding patient and appointment using unique identifiers to ensure data consistency and integrity across clinical records. These steps were implemented to ensure that only accurate, complete, and temporally consistent data were used in developing and validating the OS-B score.

The AI models utilized in this study were originally developed and trained for clinical applications, using a robust dataset that reflected diversity across key variables such as patient demographics, image quality, and sensor types. Radiographs were annotated by calibrated dentists trained through a standardized internal process. While the models were not trained specifically for this research, they were applied here in the context of oral health quantification, demonstrating adaptability to new use cases beyond their original clinical deployment.

We implemented a methodical data partitioning strategy, creating separate training and test sets with the test set comprising tens of thousands of radiographs. Both datasets maintained balanced distributions across demographic and imaging characteristics. To ensure unbiased performance evaluation, we enforced strict patient-level separation between training and test sets, preventing any single patient’s data from appearing in both.

Our development follows a continuous improvement methodology based on real-world performance feedback. We systematically monitor model performance in clinical deployment settings and analyze practitioner feedback to identify specific failure patterns or edge cases. This intelligence informs our dataset enrichment strategy, allowing us to augment both training and test datasets with representative examples of challenging scenarios. This feedback loop enables our models to progressively improve their generalization capabilities, particularly for clinically important but statistically underrepresented presentations.

### Developing the dataset

For the purposes of this study, we used deidentified data from 2,558 dental practices, which were randomly divided into three categories: a training dataset (n = 1,808), a validation dataset (n = 254) and a test dataset (n = 496). The training dataset was further subdivided to calculate a treatment probability-weighted cost-function for four clinical conditions:

1. Dental caries on teeth without crowns;
2. Recurrent dental caries on teeth with crowns;
3. Alveolar bone level and periodontal probing depth; and
4. Periapical radiolucency.

For each patient in the training dataset, we included clinical findings from their most recent dental radiographs, along with treatments provided in the 12 months following the latest radiographs as documented in the patient record using CDT codes. The average cost associated with each CDT code was calculated across all clinics. Additionally, probing depth (PD) measurements for each tooth were extracted from the patient records, with the maximum probing depth per tooth serving as an indicator of periodontal status.

Each data subset was constructed by applying filtering criteria. Initially, Overjet’s MLA determined the teeth as positive for specific findings and negative for others. Subsequently, the teeth were required to have received a specified set of treatments within one year of detecting a clinical finding being detected on a radiograph, as documented by CDT codes extracted from the patient records. Any treatments provided outside the primary dental practice were not available for inclusion in the dataset.

Table 3 provides an overview of the patient count, tooth count, along with the inclusion and exclusion criteria for the overall training dataset and subsets. For example, the caries subset includes teeth identified by Overjet’s MLA as positive for caries and negative for other clinical findings, such as margin discrepancies, calculus, root tips, bone levels exceeding 2.0 mm, PARL, implants, crowns, root tips, and bridges. Additionally, each tooth was required to have received treatment – such as a filling, crown, root canal therapy (RCT), extraction, or implant – within one year from the time of detection, as indicated by CDT codes in the patient’s electronic record, to remain in the dataset. These filtering criteria ensured that teeth included in each dataset were treated primarily due to conditions detected by Overjet AI.

**Table 3:** The number of patients, number of teeth, inclusion and exclusion criteria for the training data set and each data subset for the four specific clinical conditions.

Training data subset	Number of patients	Number of teeth	Overjet AI positive findings	Overjet AI findings exclusions	Treatments provided within 12 months of the Dental radiographs
Overall	321,530	524,298	All	Not applicable	Not applicable
Caries	292,521	454,111	Caries	crown, ABL > 2 mm, RCT, implant, bridge, PARL, calculus, root tips	Filling, crown, RCT, extraction and implants
Alveolar Bone Level (ABL) and Probing Depth (PD)	6,556	42,951	ABL > 2 mm, and the greatest (worst) PD measure for that tooth from the PMS	crown, caries, RCT, implant, bridge, PARL, calculus, root tips	Scaling and Root Planing (SRP), extraction, implants, and advanced bone level treatments
PARL	7,103	7,619	PARL	crown, ABL > 2 mm, RCT, implant, bridge, caries, calculus, root tips	RCT, extraction and implants
Crown recurrent caries	18,078	19,617	crown + caries	ABL > 2 mm, RCT, implant, bridge, calculus, root tips	Crown, extractions and implants

The inclusion and exclusion criteria in Table 3 were designed to isolate the impact of each clinical condition on treatment decisions. For each condition, criteria were selected to ensure that the treatment received was most likely attributable to that specific finding, minimizing confounding effects from co-occurring conditions. For example, in the caries subset, teeth with other significant findings—such as PARL, crowns, implants, RCTs, bridges, or alveolar bone levels greater than 2 mm—were excluded to ensure that the treatment was primarily due to caries alone. Similarly, for the PARL subset, teeth were included only if they were positive for PARL and free from other overlapping conditions that could independently influence treatment. This approach was the most effective way to reduce confounding effects when attributing treatment patterns and costs to individual clinical findings.

Table 4 summarizes patient age and gender distribution across the overall training dataset and within each data subset for the four specific clinical conditions. Patients within the caries subset were slightly younger than those in the overall training dataset. In contrast, patients with the remaining clinical conditions were older, on average, which aligns with the increased prevalence of these conditions with advancing age.

**Table 4:** Summary of patient age (median, mean, standard deviation) and gender distribution for the overall training dataset and subsets defined by four specific clinical conditions.

	Overall training dataset		Caries		Crown with recurrent caries		PARL		ABL and PD	
Median patient age	38 years		36 years		51 years		45 years		50 years	
Mean age (std. dev)	42.7 years	16.5 std. dev	40.0 years	16.0 std. dev	51.0 years	17.0 std. dev	47.0 years	17.0 std. dev	50.9 years	16.3 std. dev
Female	183,544	57.1%	166,787	57.0%	11,104	61.4%	3,972	56%	3263	49.8%
Male	132,389	41.2%	120,395	41.2%	6,806	37.7%	3,021	42.5%	3280	50.0%
Unknown	5,567	1.7%	5,308	1.8%	168	0.9%	110	1.5%	13	0.2%

### Development of a “treatment probability weighted cost-function” to calculate the OS-B tooth scores

This research uses multiple data inputs to derive a novel treatment probability-weighted cost function for determining an individual tooth score. Using tooth-specific treatments administered within 12 months after the dental radiographs and the tooth’s state as calculated by Overjet’s MLA, we developed a function to estimate treatment costs based on the tooth’s clinical condition. The tooth score is based on the treatment cost needed to restore the tooth. The scoring acknowledges that dental restorations cannot perfectly replicate original tooth health. Higher treatment costs correspond to a lower tooth score, and lower costs correspond to a higher score. Once the individual tooth scores are calculated, the patient’s OS-B is determined by averaging the tooth scores of 28 individual teeth, excluding third molars.

The treatment probability-weighted cost function integrates both the likelihood and cost of various dental treatments indicated for specific clinical conditions. The clinical state of the tooth determines a distribution of possible treatments. The estimated treatment cost is calculated by multiplying the cost of each treatment by its associated probability. Finally, this expected treatment cost is used to adjust the tooth’s health score by subtracting the weighted cost from the base score of 100 (representing a healthy tooth).

A score of 100 is assigned to a healthy tooth that exhibits no restorations or pathology. As clinical findings are detected, the score decreases accordingly. For example, a tooth exhibiting initial caries or radiolucent areas of demineralization would have a higher score than a tooth with more extensive caries requiring more invasive and expensive treatment. Conversely, a tooth with extensive caries is assigned a lower score due to the likelihood of needing a multi-surface or full coverage restoration to return it to a state of health.

To illustrate how a tooth is scored using the treatment probability-weighted cost-function, we initially focused on the caries data subset, employing the DMFP as a metric for coronal caries severity. Within our training dataset, caries emerged as the most common clinical finding, affecting 85.2% of patients and 67.3% of teeth.

**Fig. 4:**
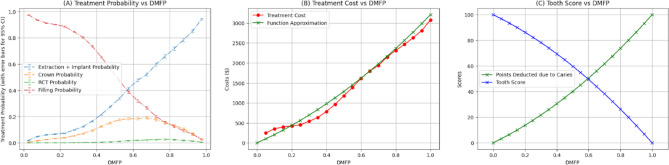
(**A**) Treatment distribution across DMFP values. The red line represents the probability of receiving a filling, which steadily declines as DMFP increases. As the DMFP value increases, the likelihood of more extensive treatments increases; the orange line shows the probability of crown placement, which peaks at a DMFP value of approximately 0.68 before declining. The green line indicates the probability of root canal therapy (RCT), which rises with DMFP. The blue line represents the combined probability of extraction or implant placement, which increases sharply with higher DMFP scores. These trends mirror clinical practice, where increasing structural damage drives a shift toward more invasive and costly interventions. The error bars in this panel illustrate the 95% confidence intervals of the computed probabilities.(**B**) Treatment cost as a function of DMFP. This plot illustrates how treatment costs escalate with increasing DMFP. The red line depicts the average observed cost of treatment within 12 months following the assessment, which increases with rising DMFP values, reflecting the need for more extensive and expensive procedures as disease severity worsens. The green line represents a second-degree polynomial approximation of cost, which closely aligns with the observed trend, validating the reliability of the prediction. (**C**) Tooth score and caries-related deductions across DMFP values. This panel displays how the OS-B score for a tooth is calculated based on its DMFP. The blue line shows the resulting tooth score, which declines from 100 to 0 as DMFP increases from 0 to 1. In contrast, the green line shows the number of points deducted due to caries, which rises proportionally with DMFP. The deduction is derived by linearly scaling the cost function in (**B**), such that no points are deducted when DMFP is 0 and the maximum 100-point deduction is applied when DMFP is 1. The opposing trajectories of the two lines highlight the scoring logic and the increasing impact of carious damage.

Figure 4A and B plot the probability of treatment and treatment cost against the DMFP value of a tooth with caries, respectively, and Fig. 4C plots tooth score as a function of DMFP. At low DMFP, the treatment cost is relatively low because only a small proportion of coronal tooth structure is compromised by demineralization or caries and a dental restoration or filling is the most performed treatment. As the DMFP increases treatment cost increases, as a larger portion of the tooth is compromised, necessitating more extensive interventions such as crowns, root canals, or extraction and placement of implants. These treatments are more invasive and expensive, leading to higher overall treatment costs.

Treatment patterns in Fig. 4A reflect clinical treatment distributions based on DMFP values. When DMFP is low—indicating minimal damage to tooth structure—fillings are the predominant treatment choice, allowing for conservative management of demineralized and carious tooth structure. As DMFP increases, showing greater structural compromise of the coronal tooth structure, treatments shift toward more extensive options like crowns, root canal therapy (RCT), or extractions with implant placement. This progression mirrors clinical practice, where severely damaged teeth require more extensive rehabilitation.

DMFP, while a valuable indicator of structural damage, is just one factor considered when making clinical treatment decisions. Clinical care is also influenced by dentist preferences, patient choices, symptoms, overall health, medical history, insurance coverage, and socioeconomic factors. For example, a patient with a high-DMFP tooth might choose extraction over a crown, RCT, or implant due to cost concerns, limited insurance, or access barriers to more complex and often more expensive treatment. This likely explains the sharp increase in extraction probability at higher DMFP values. While these external factors introduce variability in treatment selection, particularly in moderate to severe cases, the overall treatment trends consistently reflect how damage of the coronal tooth structure guides clinical decision-making in dental practice.

Figure 4B illustrates the relationship between a tooth’s DMFP and its treatment cost for the next 12 months. We approximate this relationship using a second-degree polynomial of the form (green curve in Figure 4B).

$$Cost = a*DMFP + b*{DMFP}^{2}$$

Values of *a* and *b* are obtained using the least squares regression algorithm. The tooth score is calculated by subtracting points from 100, with the deduction proportional to treatment costs over the next 12 months. This is mathematically realized by linearly scaling the polynomial via the following 2 constraints: no points are deducted when the DMFP is 0, and 100 points are deducted when the DMFP = 1.

**Fig. 5:**
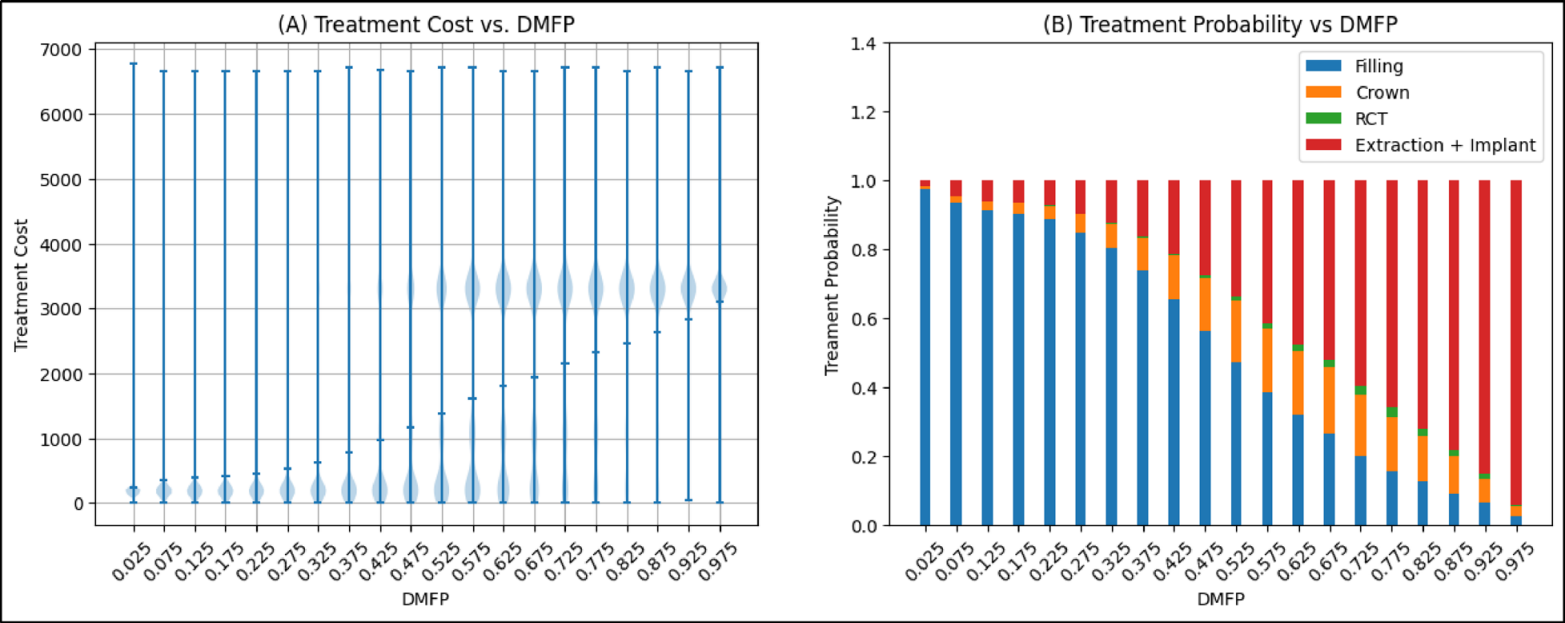
(**A**) Treatment cost distribution as a function of DMFP in the caries dataset. At each DMFP interval, the minimum, maximum and mean costs are indicated by markers ( −) at the bottom, middle, and top of each vertical bar, respectively. The blue violin plots represent the density of treatment costs, indicating the intervals where treatment costs are most frequently observed. (**B**) Stacked bar chart showing treatment probabilities across the same DMFP intervals. As DMFP increases, the likelihood of more complex treatments (crown, RCT, extraction + implant) increases, while the probability of receiving a filling decreases.

Figure 5A illustrates the variation in treatment costs as a function of DMFP. At lower DMFP values, indicating less compromised coronal tooth structure, fillings are the most common treatment, with costs ranging from $200 to $600. As DMFP increases, the likelihood of full-coverage restorations (crowns) and extractions followed by dental implant placements, also increases, resulting in higher associated costs, as shown in Figure 5B. Consequently, the cost distribution shifts upward, and for DMFP values exceeding 0.8, extraction and implant placement become the most likely treatment, with typical costs ranging from $3000 to $4000.

A tooth’s score after restoration depends on two factors: the severity of the decay and compromised coronal tooth structure and the understanding that dental treatments cannot fully restore a tooth to perfect health. Our research estimates that restored teeth regain approximately 80% of their original health status.

The severity is measured by the tooth’s Average DMFP which is defined as

$${DMFP}_{average}=\frac{{\sum }_{DMFP=0}^{1.0}P(treatment | DMFP)* DMFP}{P(treatment) }$$

Here $P(treatment | DMFP)$ denotes the probability of treatment for a given DMFP, derived from Fig. 5B. For example, the average DMFP for a crown treatment is 0.59. A tooth with this level of decay loses 50 points from its score. After crown placement, the tooth recovers 80% of these lost points, meaning only 10 points (20% of 50) are permanently deducted. This scoring system reflects that while restorative treatments significantly improve tooth function, they cannot achieve the same level of health as an original, undamaged tooth. Table 5 includes the determination of weightings for four types of restorations: 1) a full coverage restoration (crown); 2) a root canal treatment; 3) filling; and 4) an extraction and placement of a dental implant.

**Table 5:** Determination of weightings various restorations based on the tooth’s DMFP.

Restoration	Average DMFP	Points deducted due to average DMFP	Points deducted due to restoration = 0.2 * (Points deducted due to average DMFP)
Crown	0.59	50	10
RCT	0.7	61.5	12.3
Filling	0.32	[23, 50]	[4.6, 10]
Implant (extraction)	0.72	64.5	12.9

The number of points deducted for a filling depends on its size, with a minimum deduction of 4.6 points and a maximum of 10 points. Here we capped the point deductions for fillings to that of a crown treatment because filling treatments generally retain more original coronal tooth structure as compared to a crown.

To account for findings such as PARL and recurrent caries under crowns, we used a simple weighted average technique to determine point deductions. For each of these conditions we obtained the probability distribution of different treatment types, and used the DMFP-based point deduction for each of those treatments together with the probabilities as the weights, to find the average point deductions. Table 6 provides a summary of the treatment distributions and corresponding point deductions for PARL and recurrent caries under crown restorations, as represented by the following formula:

$${TS}_{condition}= \sum\limits_{t=1}^{n}P(t){TS}_{t}$$

where  ${TS}_{condition}$ represents either PARL or recurrent caries under crown, $P(t)$ denotes the probability of a given treatment for the condition, and  ${TS}_{t}$ is the DMFP-based point deduction for the treatment performed for the condition.

**Table 6:** Summary of treatment distribution and tooth score point deductions for PARL and recurrent caries associated with a crown restoration.

Condition	Root canal (RCT)	Crown	Extraction + implant	Points deduction
PARL	59%	–	41%	63.3
Crown recurrent caries	–	82%	18%	52.6

**Fig. 6:**
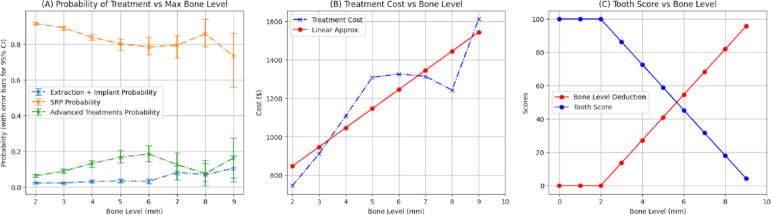
(**A**) Probability of various treatment types based on the bone level of teeth in the AL & PD dataset. The error bars in this panel illustrate the 95% confidence intervals of the computed probabilities. Advanced treatments may include procedures such as gingival flap surgery, osseous surgery, bone replacement grafts, and distal or proximal wedge procedures. (**B**) Relationship between treatment cost and bone level for the same set of teeth. The blue curve represents the estimated treatment costs, while the red line shows a first-degree polynomial approximation of cost. (**C**) Tooth Score vs. Bone Level. Tooth score decreases linearly as bone level exceeds 2.0 mm, with deductions scaled to treatment cost (**B**). At 6.71 mm, the average extraction threshold, 63.5 points are deducted. This scoring approach aligns with caries-based indices for consistent assessment of tooth health.

Points are deducted when a tooth’s bone levels exceed 2.0 mm, where a measurement ≤ 2.0 mm is considered healthy. The deduction amount is proportional to the treatment cost at that bone level. Figures 6A and B illustrate the treatment probability and associated costs over the next 12 months as a function of a tooth’s bone level.

Following a methodology similar to that used in caries analysis, a first-degree polynomial is used to approximate the relationship between treatment cost and bone level of a tooth (red curve in Figure 6B):

$$Cost = a*BL + b$$

The values of $a$ and $b$ are determined using the least squares regression algorithm. This first-order polynomial is then linearly scaled based on two constraints: no points are deducted when the bone level is less than or equal to 2.0 mm, and 63.5 points are deducted when the bone level reaches 6.71 mm. Similar to the Average DMFP, the Average Bone Level ($BL$) for extraction is 6.71 mm. We propose that the tooth score for a tooth requiring implant placement and restoration whether due to elevated bone levels or severe caries, should be equivalent. Figure 6C plots tooth score as a function of bone level.

Interproximal calculus on a tooth’s cementum typically requires scaling and root planing (SRP) treatment, with an associated cost equal to that of a tooth displaying a DMFP of 0.07, as seen in Figure 4B. According to the relationship between DMFP and tooth score (Figure 4C), 4.5 points are deducted from a score of 100 at this DMFP. Therefore, the presence of interproximal calculus results in a 4.5-point deduction. Similarly, a tooth typically requires SRP treatment when its probing depth exceeds 4 mm. Following the same point deduction approach as for interproximal calculus, 4.5 points are deducted when the probing depth surpasses 4 mm.

Point deductions due to Margin Discrepancy (MD) vary based on its type. If the margin discrepancy occurs on a filling, the deduction is based on the tooth’s DMFP. If the MD occurs on a crown, we assume that the tooth requires crown replacement, leading to a deduction of 50 points. A deduction of 100 points is applied when a tooth is missing or when only a root tip remains. Table 7 summarizes the point deductions for each clinical condition.

**Table 7:** Summary of point deductions for each clinical condition.

Condition	Points deduction
Missing tooth	100
Root tip	100
PARL	63.3
Crown recurrent caries	52.6
Caries	60.41*DMFP + 39.59*DMFP2
Bone Level (BL)	13.67*BL—27.33
PD > 4 mm or Interproximal calculus on cementum	4.5
Margin discrepancy (on filling)	(60.41*DMFP + 39.59*DMFP2)
Margin discrepancy (on crown)	50
Filling	[4.6, 10]
Crown	10
RCT	12.3
Implant	12.9

**Fig. 7:**
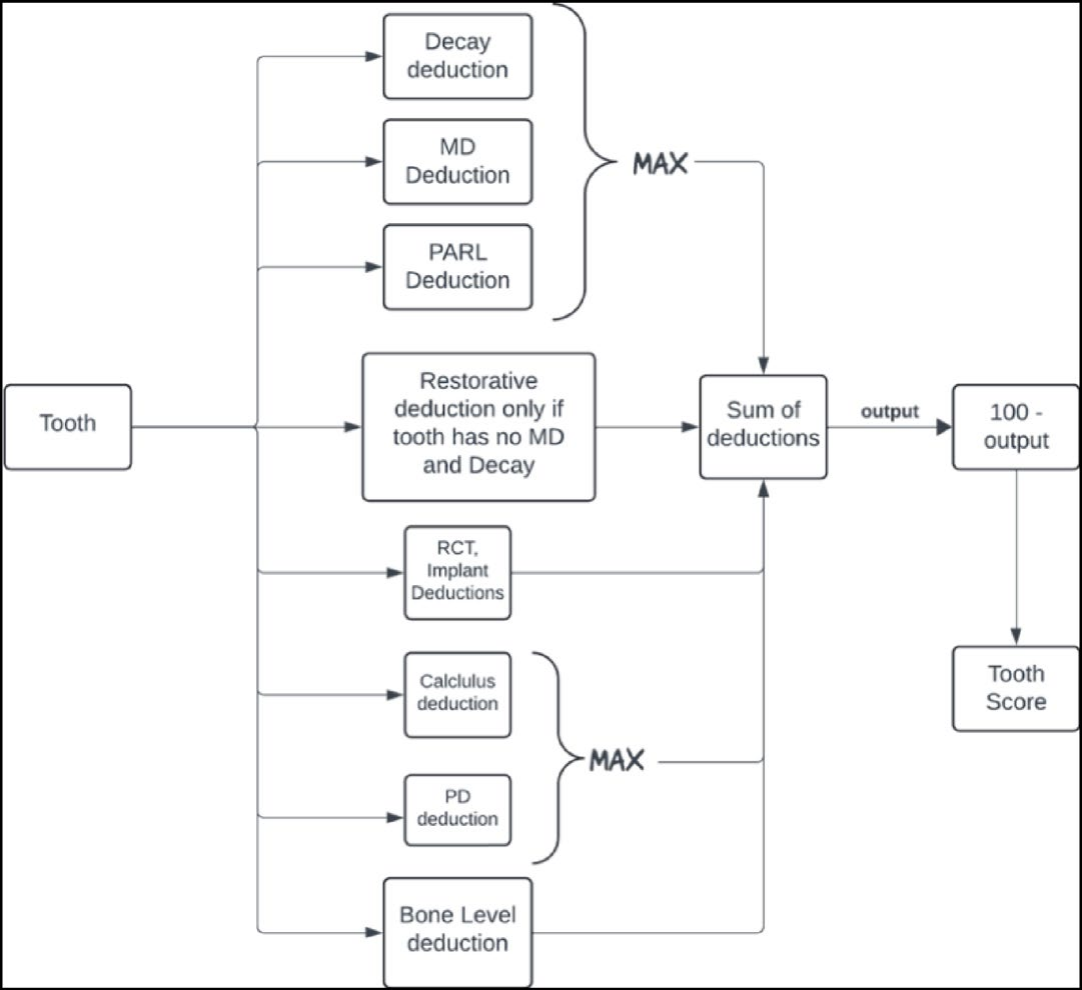
OS-B is calculated by first determining individual tooth scores, as shown in this figure, and then averaging these scores across the 28 permanent teeth, excluding third molars. Missing teeth and root tips are assigned a score of zero.

The previous sections explored how each of the eight clinical findings affects individual tooth scores. Each individual finding results in a specific number of points deducted from an ideal score of 100. When multiple findings are present, each deduction is calculated separately and then they are combined, as illustrated in Figure 7. The total deduction is subtracted from 100 to yield the final tooth score, while missing teeth and root tips are automatically assigned a score of zero.

**Fig. 8:**
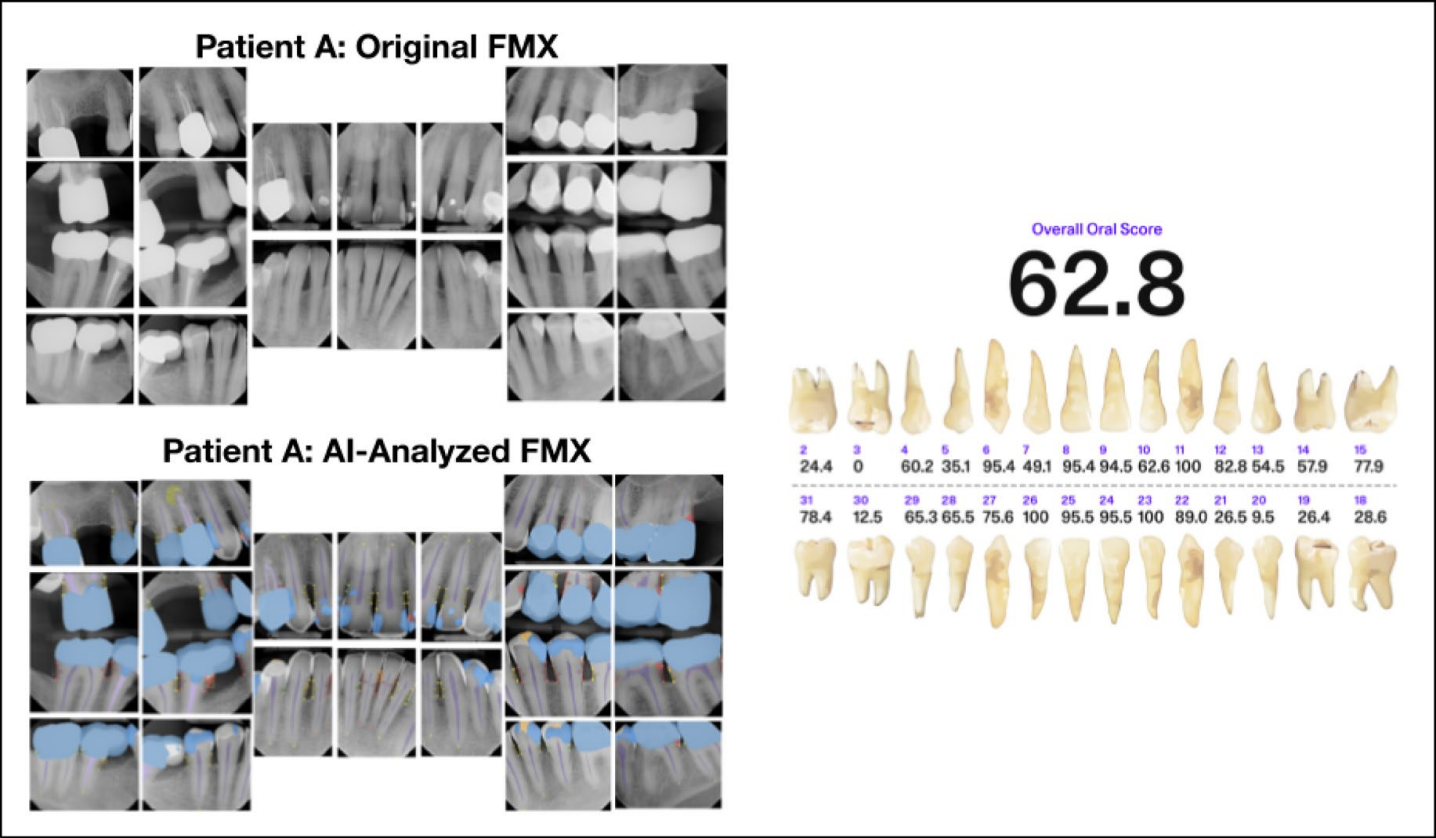
Patient A’s original full-mouth X-rays (FMX) without AI predictions can be compared to the AI-analyzed FMX. Using AI predictions and the calculated oral score for each tooth, the odontogram provides individual tooth scores and an overall oral score. Patient A has an overall Oral Score of 65.5, impacted by findings including PARLs, bone levels, caries, calculus, MD, RCTs, and extensive restorative treatments.

**Fig. 9:**
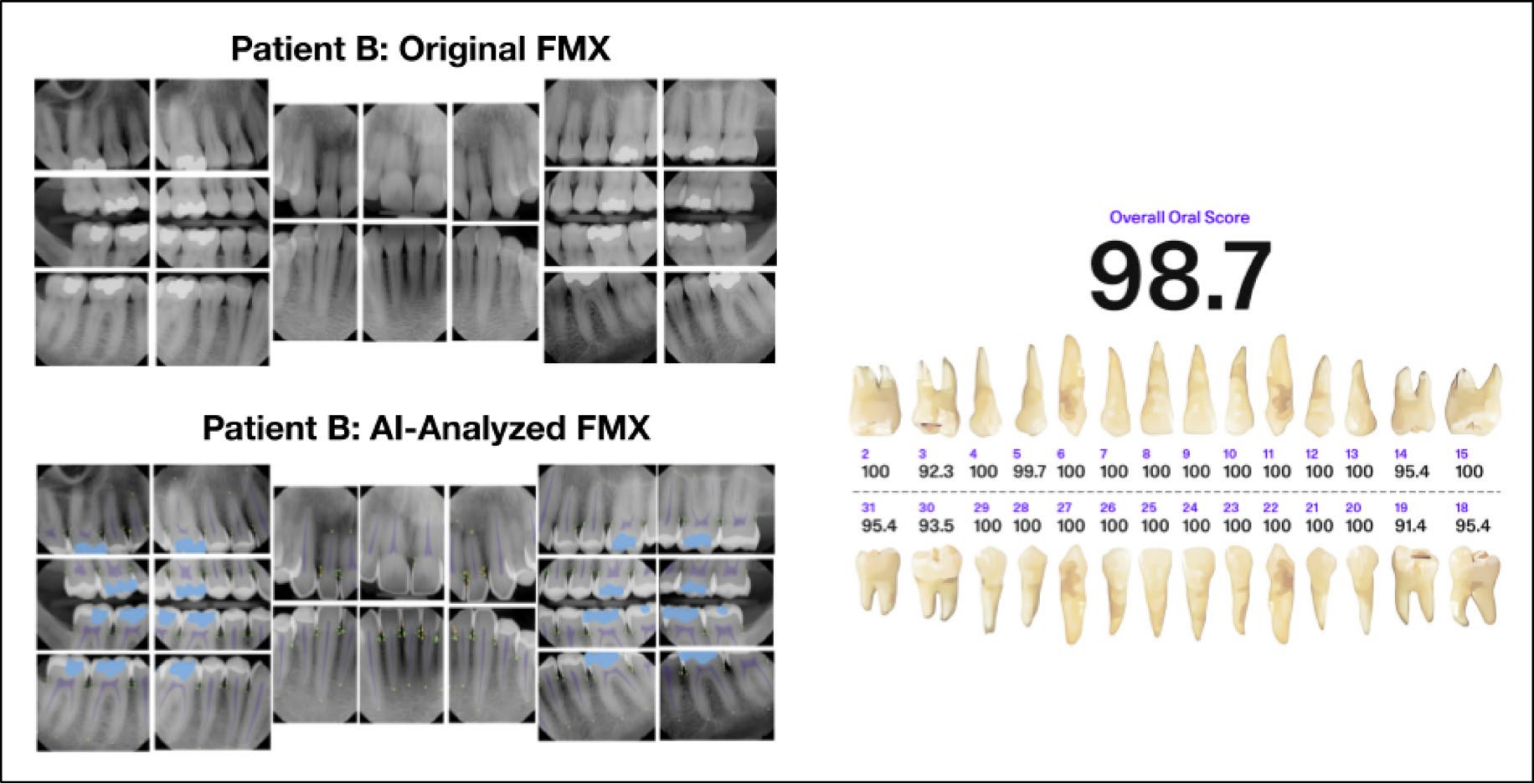
Patient B’s original FMX and AI-analyzed FMX illustrate predications used to calculate each tooth’s Oral Score. The odontogram indicates an overall oral score of 98.7, primarily attributed to six restorations. This patient most likely has a prior history of dental decay that was successfully treated with dental restorations, restoring the patient’s health to an improved but not perfect OS-B of 98.7.

Since multiple conditions can often be addressed with a single restorative or endodontic procedure, treatment costs are non-additive. Thus, deductions for decay, MD, and PARL are combined by taking the maximum value among these findings. Similarly, deductions for elevated probing depth and interproximal calculus are also combined using the maximum value, as both conditions are typically treated together through SRP. Bone level deductions are treated independently from other findings, reflecting their distinct nature and specific treatment requirements. Restorative deductions (crowns and fillings) are only applied if there is no concurrent MD or decay, as restorations are automatically accounted for by the DMFP when these conditions are present. Figure 7 provides an illustration of the calculation process for individual tooth scores, while Figures 8 and 9 demonstrate the application of these calculations in patient cases.

*Example: Step-by-step calculation of individual tooth score*

We provide the following example to illustrate how individual tooth scores are calculated using our methodology. Consider a tooth with the following clinical findings:

- Moderate decay affecting 40% of the coronal area (DMFP = 0.4)
- Radiographic interproximal bone level measurement of 4.0 mm
- Periapical radiolucency (PARL)
- Calculus present on the root surface

*Step 1.* Calculate individual condition deductions, approximated to one decimal place.

- Caries deduction: DMFP of 0.4 results in 30.5 points deducted using the equation from Table 7: (60.4 × 0.4) + (39.6 × 0.4^2^) = 24.2 + 6.3 = 30.5 points
- Bone loss deduction: 4.0 mm bone level results in 27.4 points deducted: (13.7 × 4.0) − 27.3 = 54.8 − 27.3 = 27.5 points
- PARL deduction: 63.3 points (fixed value from Table 7)
- Calculus deduction: 4.5 points (fixed value from Table 7)

*Step 2.* Apply clinical prioritization rules As illustrated in Fig. 7, the scoring system applies clinical rules to avoid double-counting related conditions:

- Decay vs. PARL: Use the maximum deduction (63.3 points for PARL > 30.5 points for caries)
- Bone loss: Applied independently (27.5 points)
- Calculus: Applied independently (4.5 points)

*Step 3.* Calculate final tooth score. Final calculation: 100 − (63.3 + 27.5 + 4.5) = 4.7

This tooth score of 4.7 out of 100 indicates the severely compromised condition of the tooth requiring extensive treatment.

## Results

The dataset and subsets developed for this study are large, geographically dispersed, and generally represent the population of patients who seek care at dental practices across the United States. There are slightly more females than males, which is expected because females have slightly higher annual dental visit rates as compared to males. For example, the 2020 National Health Interview Survey (NHIS) indicates that 69.4% of females visit the dentist annually as compared to 64.2% of males^17^. The distribution of clinical findings as seen in Table 1 approximate epidemiological studies of the prevalence of these conditions^18^.

### Correlation of OS-B with tooth treatment cost

Prior work by Marcus et al.^3^ to develop an Oral Health Status Index (OHSI) used a paired preference technique and data from 232 simulated adult patient cases to create 315 pairs; 12 dentists were asked to choose the healthier patient in each pair. This information was then used to determine weights for each clinical finding. The scores of all 32 teeth were summed to generate the overall oral score.

We compared the two dental scoring systems, the OHSI tooth level score and the new OS-B tooth score, by examining how well they predict future treatment costs. We analyzed data from 124,583 teeth across 36,164 patients in 454 clinics not involved in OS-B's development. The study used CDT codes to determine treatment provided within that dental practice within 12 months of the date of the dental radiographs.

We calculated both OHSI and OS-B scores for each tooth and compared them to treatment costs using Pearson correlation coefficients^19^:

- OHSI Score: − 0.134
- OS-B Score: − 0.441

The negative correlations indicate that healthier teeth (higher scores) require less expensive treatments. OS-B showed significantly stronger predictive power (-0.441) compared to OHSI (-0.134), representing a 200% improvement. This improved accuracy stems from OS-B's ability to account for disease severity. For instance, while OHSI deducts the same 2.4 points for both minor and severe cavities, OS-B assigns different scores based on caries severity as measured by the DMFP, with the understanding that more severe cavities result in higher treatment costs.

### Impact analysis of clinical findings

OS-B evaluates tooth health using nine clinical findings, each weighted differently to calculate the final tooth score. To understand the importance of each finding, we performed a leave-one-out analysis, removing one component at a time and measuring how this affects the score’s ability to predict future treatment costs.

Results (correlation with future treatment costs):

- Complete OS-B Score: − 0.44
- Without Caries: − 0.22
- Without Bone Loss: − 0.45
- Without PARL: − 0.44
- Without Restorations: − 0.42

Removing the caries component caused the most significant drop in predictive power (from -0.44 to -0.22). This makes sense clinically as caries is a common, treatable condition that often requires expensive procedures (fillings, root canals, extractions and implants). In contrast, removing other components had minimal impact. Bone loss, for example, barely affected the correlation (-0.45). Similarly, existing restorations without active disease (-0.42) typically do not need immediate treatment. This suggests that caries status is a dominant driver of near-term treatment needs and costs. Given its strong influence on predictive accuracy, future work could explore whether assigning greater weight to the caries component in the OS-B scoring system may further enhance its clinical utility. Such refinements, coupled with validation across diverse datasets, could improve the model’s ability to guide both practitioners and patients toward more timely and cost-effective interventions.

### OS-B scores: age and gender patterns

**Fig. 10:**
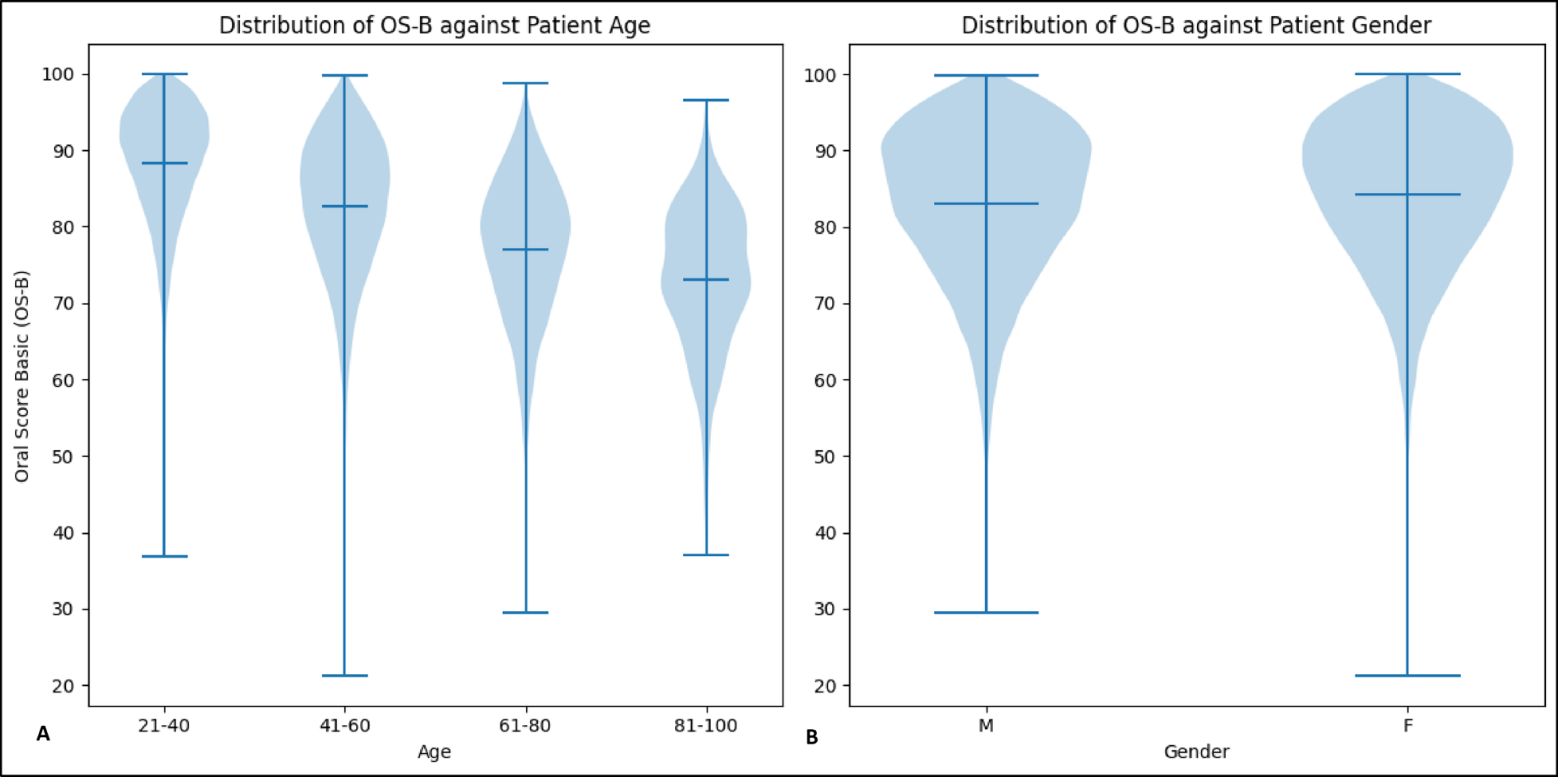
OS-B distribution analysis. (**A**) Shows the distribution of Oral Score Basic (OS-B) across four age groups: 21–40, 41–60, 61–80, and 81–100. Each age group has a violin shape representing the distribution of OS-B scores, with a mean line and 2 lines showing maximum and minimum. (**B**) Shows the distribution of OS-B scores for male (M) and female (F) patients. Each gender has a violin shape showing the range of OS-B scores, with a line indicating the mean and others for the maximum and minimum scores.

Analysis of OS-B scores demonstrates predictable patterns across age and gender demographics. As expected, oral health scores progressively decline with age, reflecting the cumulative impact of dental diseases over time. Gender-based analysis reveals a consistent pattern where women maintain marginally higher OS-B scores compared to men across all age groups. This gender disparity aligns with established national health data, which documents men’s increased susceptibility to oral health challenges, including higher rates of periodontal disease, oral cancer, and dental trauma, often attributed to less rigorous oral hygiene practices and fewer dental visits^20^. These demographic trends in OS-B scores are visually represented in Figure 10.

## Discussion

Our study represents a significant advancement in oral health assessment through the application of artificial intelligence and computer vision to analyze radiographic and clinical data from 2,558 U.S. dental practices. The novel treatment probability-weighted cost function provides a more sophisticated approach to quantifying oral health compared to previous methodologies. The OS-B addresses key limitations of previous scoring systems, notably the Oral Health Status Index^3^. While OHSI was valuable, its development was constrained by limited clinical examiners and sample size. Additionally, OHSI’s binary categorization of complex conditions like dental caries failed to capture disease severity, which is a crucial determinant of treatment needs and costs. Our validation demonstrates OS-B's superior predictive power for future treatment costs (correlation coefficient −0.44 versus −0.13 for OHSI), representing a 200% improvement. Clinically, this significant enhancement translates into a more accurate estimation of treatment costs over the following 12 months. Unlike the OHSI, OS-B more effectively highlights the urgency and value of preventive care and early interventions. By offering a score that patients can easily interpret, it supports more informed health decisions and encourages behaviors aimed at maintaining a higher score, ultimately reducing long-term treatment needs and associated costs.

The OS-B offers value to multiple stakeholders: clinicians, payers, and consumers. For clinicians, the OS-B represents the first step toward an efficient, standardized, and objective metric for measuring dental status and clinical outcomes. The OS-B provides clinicians with a simple, objective, and consistent way to assess the health of individual teeth. By integrating radiographic findings with probing depth measurements, it helps standardize how tooth conditions are evaluated across different patients, providers, and visits. This makes it easier to track changes over time, assess risk, and plan personalized treatments based on the patient’s needs. For payers, future iterations of this methodology would enable the development of data-driven quality metrics that can be used to assess the impact of clinical interventions, programs, and plan designs on patient oral health.

Additionally, a consumer version could be developed to help individuals better understand their oral health status, identify improvements for daily oral care, and recognize the potential benefits of professional interventions. A consumer-friendly version of the OS-B score has the potential to significantly increase patient engagement by making oral health more tangible and trackable. For it to be effective, such an application would need to be easily accessible—ideally via a smartphone app that patients can interact with on a daily basis. The score would update automatically after each dental visit, incorporating the latest clinical data. In addition to clinical findings, the consumer version could also factor in self-reported behaviors such as brushing and flossing frequency, dietary habits, and lifestyle factors. By providing users with a regularly updated, easy-to-understand metric—similar to a sleep or fitness score—patients may become more proactive about their oral hygiene. This type of feedback loop can reinforce healthy behaviors and encourage timely dental visits, ultimately supporting preventive care and better long-term oral health outcomes.

Further research is needed to further validate the OS-B and to evaluate the addition of other oral health components to expand its utility and enable a more holistic assessment of oral health. Impact analysis identified dental caries as the strongest predictor of future treatment costs, affecting 85.2% of patients and 67.3% of teeth in our dataset. However, this finding may partially reflect methodological constraints in periodontal assessment, which was limited to interproximal bone levels and pocket depth measurements. The OS-B demonstrated expected demographic trends across age and gender, aligning with established epidemiological patterns. However, several limitations warrant acknowledgment, including the reliance on radiographic findings from patients with dental visits as well as a limited number of variables that do not fully capture the complexity of periodontal disease. Further research is needed to assess the incremental value of including a more robust set of periodontal measures such as clinical attachment loss, bleeding on probing, and furcation involvement to the calculation of the OS-B.

The cost-based weighting considers CDT codes for care that was delivered to each patient. However, we do not take into account care that was recommended and not provided, nor do we know why that treatment was not completed. We also did not consider any dental care that was provided by a dental specialist or other dental practitioner beyond the practice data available for investigation. However, because the dataset is derived from many dental practices across the U.S., it is likely to be representative of general dental care provided to patients in the U.S. as compared to studies that include a smaller number of patients or care provided by a more limited panel of clinicians.

Overjet currently serves more than 2,500 dental clinics across the United States, with our OS-B development and validation dataset derived from this clinical network. While this provides a robust foundation for U.S. population assessment, we acknowledge important limitations in demographic representation and geographic diversity—particularly regarding populations outside the U.S. Further research should address these limitations through clinical validation using data sets with patients located both within and beyond the U.S. These combined efforts will enable us to quantify OS-B performance across different populations, identify necessary adjustments to account for regional oral health variations, and ultimately refine the OS-B formulation to ensure meaningful applicability across global dental care settings.

While the OS-B represents a significant advancement, it is limited by its reliance on radiographic findings from patients with dental visits and limited periodontal measures and does not account for soft tissue conditions, measures of oral function or other patient-reported oral health measures. The OS-B does not account for variations in treatment planning and the nuanced process of prioritizing treatment delivery, as well as patient treatment acceptance. This research focused on adult patients and was not intended to be applicable to dental patients under the age of 21 years. Future iterations should aim to incorporate these factors, be extended to other age groups, and undergo additional clinical validation in various patient groups or populations. This research should also be expanded to focus on risk indicators, including bio-behavioral variables as well as information about the patient’s medical conditions and medications. Future research should also explore the relationship of the score to dental practice type, as well as to additional provider and patient characteristics including social determinants of health.

We recognize that the use of AI in dentistry raises important ethical considerations, particularly regarding its role in clinical decision-making, the potential for bias and concerns for data privacy. A detailed discussion of ethics, bias, privacy, and provider oversight is beyond the scope of this manuscript. However, a recent report from the U.S. National Academy of Medicine presents an AI Code of Conduct framework to guide the responsible development and use of AI in health and medicine based on a set of principles^21^. The report encourages that all stakeholders play a role in ensuring that “health AI contributes positively to society and advances in the human condition, and avoids the risks associated with incongruent or malicious use of the tools and technologies.” Similarly, the World Health Organization has published guidance on ethics and governance of AI for health^22^ and many professional organizations have done the same, providing guidance for specific health disciplines. Two notable publications focused on the use of AI in dentistry include a Standards Committee on Dental Informatics White Paper from the American Dental Association^23^ and the FDI World Dental Federation policy statement^24^.

Recognizing the potential for bias in AI systems—particularly those trained on imbalanced datasets—Overjet proactively builds and curates large and diverse training datasets that reflect a wide distribution of patient age, gender, and image acquisition characteristics. The same principles were applied in the development of the OS-B score. Further research and validation of the OS-B should include the use of non-U.S. data sets as well as data sets with broad representation in terms of geographic regions and sociocultural factors. Overjet is committed to incorporating data from a broader range of patients to further strengthen the fairness and generalizability of our models. Adhering to the highest standards of patient care and privacy protection, we maintain strict HIPAA compliance and implement sophisticated security protocols across our systems, further validated by our HITRUST certification. Regular monitoring and updates maintain these high standards, and rigorous testing and regulatory clearance precede any deployment in patient care.

In clinical practice, our FDA-cleared dental AI technologies function exclusively as supportive tools for licensed dental professionals, enhancing their diagnostic capabilities while preserving the fundamental importance of clinical expertise and judgment. Overjet’s AI technologies, including the models supporting the OS-B score, are designed to function strictly as assistive tools to aid licensed dental professionals in diagnosis and treatment planning. They are not intended to replace clinical judgment or to independently determine patient care decisions. Overjet’s dental AI products have received FDA clearance specifically for use in this assistive capacity.

The OS-B presented in this research, while limited to radiographs and probing depth measurements, provides a pathway for incorporating patient data from various sources and modalities, including extraoral and intraoral photographs, Cone-Beam Computed Tomography (CBCT) and other dental imaging modalities, cephalometric analysis, medical history, and other risk indicators including salivary biometrics into the formulation of an oral health score. In the future, AI capabilities in dentistry will extend far beyond radiographic interpretation, advancing toward a multi-modal representation of patients’ oral health. The development of the next generation of multi-modal AI in dentistry will help address the limitations of the current OS-B and pave the way for an advanced and more comprehensive version of the oral score.

## Conclusion

To the best of our knowledge, OS-B represents the first large-scale data-driven approach to summarize the health status of individual teeth as well as provide a patient-level summary score. Our approach leverages dental healthcare costs as an objective measure to quantify the severity of various conditions, which were incorporated into the current definition of OS-B. Except for probing depth measurements, OS-B can be automatically calculated based on a detailed analysis of patients’ dental radiographs using the Overjet AI platform. OS-B shows good trends at the population level such as decreasing with age, showing some differences between men and women. Our approach of using treatment cost for each tooth as a basis paves the way to an oral score with multiple potential applications and benefits. We strongly believe that the robust evidence presented in this research suggests that AI and large-scale data will profoundly impact the improvement of oral health, with tools like the OS-B playing a pivotal role in centering care around the patient.

## Data Availability

The dataset supporting the findings of this study will be made available to qualified researchers upon reasonable request, at no cost, following publication. Interested parties may submit a request for access to ResearchRequest@Overjet.ai. Upon approval, researchers will be provided with a secure download link for receiving the data. In no event will any protected health information be made available to researchers. Receipt of the data will be subject to Overjet’s terms and conditions.
